# Recent Progress in Nanomaterial-Based Fluorescence Assays for the Detection of Food-Borne Pathogens

**DOI:** 10.3390/s24237715

**Published:** 2024-12-02

**Authors:** Shiyu Song, Lu Han, Min Chen, Leiqing Pan, Kang Tu

**Affiliations:** 1 College of Food Science and Technology, Nanjing Agricultural University, Nanjing 210095, China; 9201810119@stu.njau.edu.cn (S.S.);; 2 College of Ocean Food and Biological Engineering, Jimei University, Xiamen 361021, China

**Keywords:** food-borne pathogens, fluorescence materials, fluorescence assay, detection

## Abstract

Food safety is of great concern, and food-borne bacterial infections and diseases are a major crisis for health. Therefore, it is necessary to develop rapid detection techniques for the prevention and recognition of food safety hazards caused by food-borne pathogens. In recent years, the fluorescence assay has become a widely utilized detection method due to its good signal amplification effect, high detection sensitivity, high stability, and short detection time. In this review, the different kinds of fluorescence materials were concentrated, including quantum dots (QDs), carbon dots (CDs), metal–organic frameworks (MOFs), and upconversion nanoparticles (UCNPs). The optical properties and applications of different kinds of fluorescent materials were analyzed and compared. Furthermore, according to the biosensing components, different fluorescence biosensors are reviewed, including label-free based fluorescence probes, aptamer-based biosensors, and antibody-based biosensors. Finally, we focused our attention on the discussion of fluorescent detection techniques combined with other techniques and their applications. The review presents future trends in fluorescence sensors, providing new sights for the detection of food-borne pathogens.

## 1. Introduction

Food-borne diseases, as one of the significant public health concerns worldwide, pose a serious threat to the health of humans. Differing from infectious diseases, food-borne diseases are illnesses that result from the consumption of contaminated food [1]. There are various types of food-borne pathogens, and they can be divided into infectious and toxin-producing types according to their biological characteristics. Infectious pathogens include pathogenic *Escherichia coli* [2], *Vibrio parahaemolyticus* [3], *Listeria monocytogenes* [4], etc., while toxin-producing pathogens include *Staphylococcus aureus* [5], *salmonella* [6], *Clostridium botulinum*, *Bacillus cereus*, etc. Although the clinical symptoms of food-borne diseases are mild and self-healing [7], they are still a significant problem due to the large number of people affected each year [8]. According to the World Health Organization, about 10% of people worldwide fall ill each year due to consuming contaminated food [9], which puts a strain on healthcare systems and undermines national economies, tourism, and trade, thereby hindering socio-economic development.

Therefore, developing a rapid detection method to avoid the consumption of foods contaminated with pathogens is necessary. Traditional detection techniques for food-borne pathogens mainly include plate counting [10], molecular biological detection technology [11,12,13,14], and immunodetection technology [15,16]. Currently, the traditional culture-based method is still the gold standard method with its high accuracy and sensitivity. However, the above methods for food-borne pathogen detection have been restrained due to short shelf life, the need for large sample volumes, and lengthy processing times. With the development of nanotechnology and molecular technology, many new rapid detection methods have been proposed and applied to the detection of food-borne pathogens.

These new detection methods mainly include the colorimetric method [17,18,19], electrochemical method [20,21], Raman detection method [22,23], and fluorescence detection method. In contrast to the traditional culture-based methods, the above detection methods can enable the generation of fast and reliable results for ensuring food safety. The performance of these rapid detection methods can be noticed from some research work for food-borne pathogen detection. Additionally, due to the complexity of the food matrix, these detection methods have some limitations, respectively. A list of the detailed detection methods for food-borne pathogens, including assay time, detection limit, potential limitations, and potential limitations, is given in Table 1.

Among all novel detection methods, fluorescence assays become a widely used detection method due to high detection sensitivity, high stability, and short detection time [36]. They are a quantitative analysis method and build a linear relationship between fluorescence intensity and the concentration of the detection target, and they meet the requirement of being fast, highly sensitive, and selective.

In this review, we provide a comprehensive discussion of recent developments in fluorescence assays based on different specific recognitions for food-borne pathogen detection by showing the latest materials, principles, and properties of fluorescence assays. In addition, we review the current advances in fluorescence detection applications for other targets, offering perspectives and insights for food-borne detection in the future.

## 2. Fluorescence Materials

The core of fluorescence assays for food-borne pathogen detection can be divided into two parts: fluorescent material and the component of specific recognition. Traditional fluorescent materials such as rhodamine dyes [37], cyanine dyes [38], and Alexa dyes [39] have been successfully applied in the food safety field. However, fluorescent dyes have many defects, such as high background signals, a narrow excitation spectrum, a broad and asymmetric fluorescence emission spectrum, and susceptibility to photobleaching [40], which significantly restrict their application in the field of food safety.

Nowadays, novel fluorescence materials for detecting food-borne pathogens such as quantum dots, metal–organic frameworks, and upconversion nanoparticles are gradually replacing traditional fluorescent materials due to their advantages. These new materials offer several advantages, including small size, strong adsorption capacity, and high surface reactivity, which enables them to bind with a lot of bacteria at the same time, resulting in a higher fluorescence intensity [41]. Table 2 shows the strategies for food-borne pathogen detection based on different fluorescence materials, the limit of detection (LOD), detection time, and detection samples for bacterial detection. In addition, the optical properties and applications of different kinds of fluorescence materials are analyzed and compared.

### 2.1. Quantum Dots

Quantum dots (QDs) are emerging nanomaterials in recent years with particle sizes of 1–10 nm [55]. Due to their unique optical properties, they have become very promising fluorescent nanomaterials. In addition, the doped QDs obtained by doping different materials will have the luminescence properties inherent in QDs and the luminescence properties of doped ions. The interaction between QDs and intended pathogens caused fluorescence quenching or enhancement in QDs [56]. The main mechanisms include fluorescence resonance energy transfer (FRET), photoinduced electron transfer, internal filtration effects, aggregate effects, static quenching effects, and dynamic quenching effects [57].

**Figure 1:**
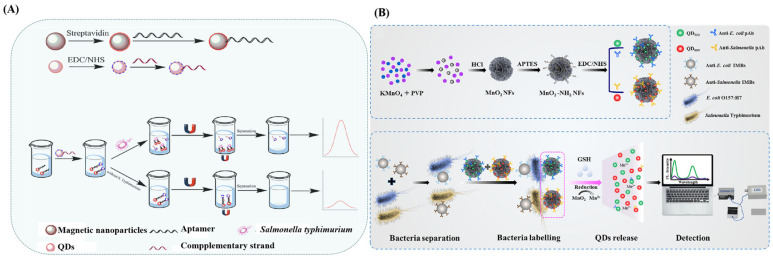
(**A**) The strategy using Fe_3_O_4_ magnetic separation and CdTe quantum dots for fluorescence detection of *Salmonella*. (**B**) The strategy for *E. coli* and *Salmonella* detection using MnO_2_ NFs combined with QDs. (**A**) Adapted from Ref. [42]; (**B**) adapted with permission from Ref. [43]. Copyright 2024 Elsevier.

Ren et al. reported a fluorescence assay leveraging the high sensitivity and stable fluorescence of CdTe QDs combined with a specific DNA aptamer for the detection of *S. typhimurium* [42] (Figure 1A). In their research, aptamer-coated magnetic particles (Apt-MNPs) were employed as target captors, while the CdTe QD-labeled complementary strands served as signal generators. The fluorescence of CdTe QDs increases linearly in the concentration range of 10 to 1010 CFU/mL, with a detection limit of 1 CFU/mL. Xue et al. developed a fluorescent sensor for the simultaneous detection of *E. coli* O157: H7 and Salmonella typhimurium using immunomagnetic nanobeads (MNBs), manganese dioxide nanoflowers (MnO_2_ NFs), and QDs [43] (Figure 1B). QDs@MnO_2_ nanocomposites were obtained with MnO_2_ NFs and QDs, followed by modification with antibodies (pAbs) to obtain pAb-QDs@MnO_2_ nanocomposites (QM NCs). The target bacteria were first conjugated to MNBs and QM NCs to create the MNB–bacteria–QM complex. Glutathione is then used to reduce MnO_2_ to Mn^2+^, rapidly releasing QDs from the complex. This assay enables simultaneous quantification of *E. coli* and Salmonella within 2 h with detection limits of 15 CFU/mL and 40 CFU/mL, respectively. The above examples show the potential of quantum dots in food-borne disease detection. However, the potential toxicity to humans and the environment and the high cost of quantum dots are key issues to be considered in future research.

Although much research has been conducted on QD-based fluorescent nanosensors, many challenges remain, including reducing their toxicity, enhancing their chemical stability, repeatability, high uniformity, and fluorescence performance. Future research should focus on the selection of appropriate doping materials, the optimization of the synthesis, and the doping process of QDs [58].

### 2.2. Carbon Dots

Carbon dots (CDs) are an emerging fluorescent material that have garnered significant interest as alternatives to conventional QDs [59]. CDs possess several advantages, including excellent biocompatibility, low toxicity, high water stability, and ease of synthesis [60]. Moreover, their customizable surface functional groups and fluorescence properties make them highly suitable for sensing and detection [61]. CDs feature many hydroxyl and carboxyl groups on their surface, enabling them to conjugate with various biomolecules through these functional groups [44]. During interaction with bacterial cells or their metabolic products, their photoluminescent qualities allow for the sensitive identification of bacteria on the basis of either quenched or enhanced fluorescence [62].

**Figure 2:**
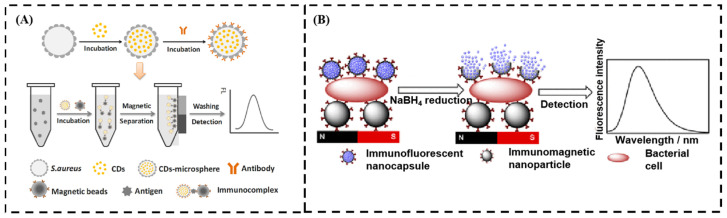
(**A**) Strategy for the development of cell-based CD microspheres with *S. aureus* cells and their application as a fluorescent immunosensor for pathogen detection. (**B**) Illustration of the detection of *S. aureus* based on CDs@BONs. (**A**) Adapted with permission from Ref. [63]. Copyright 2021 Elsevier; (**B**) adapted with permission from Ref. [45]. Copyright 2018 American Chemical Society.

Zhao et al. developed a highly sensitive fluorescent immunosensor for detecting *E. coli* O157:H7 using microspheres labeled with CDs [63]. In this study, CD microspheres were prepared using *S. aureus* cells as the carrier to incorporate CD particles (Figure 2A). The microsphere can be easily combined with various antibodies. When combined with the immunomagnetic bead techniques, the CD microsphere immunosensor was established for the specific detection of *E. coli* O157:H7. Yang et al. prepared CD-encapsulated breakable organosilica nanocapsules (BONs) as advanced fluorescent labels for the ultrasensitive detection of *S. aureus* [45]. The CDs are entrapped in organosilica shells to form core–shell CDs@BONs (Figure 2B). These fluorescent nanocapsules are then conjugated with anti-*S. aureus* antibody to specifically recognize *S. aureus*. Compared with conventional immunoassays using CDs as fluorescent labels, the fluorescent signals are amplified by two orders of magnitude due to the presence of hundreds of CDs encapsulated in each nanocapsule.

In addition, CDs have also been widely used in detecting *S. typhimurium* [59], Helicobacter pylori [46], Salmonella [47], etc.

### 2.3. Metal–Organic Framework

Metal–organic frameworks (MOFs) are organic–inorganic hybrid crystalline materials formed by metal ions or clusters and organic ligands via coordination bonding [64]. The special structure endows MOFs with many advantages, including high specific surface area, controllable pore structures, and significant thermal stability [65], which make MOFs a potential material in food-borne pathogen detection.

**Figure 3:**
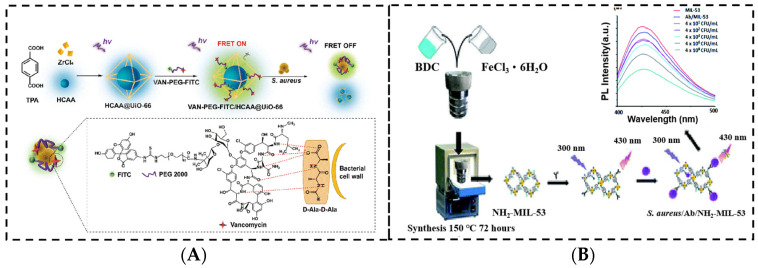
(**A**) Schematic interpretation of the metal–organic framework-based fluorescence resonance energy transfer nanoprobe. (**B**) Fluorescent assay for *S. aureus* detection using an antibody/metal–organic framework bioconjugate. (**A**) Reproduced from Ref. [48] with permission from the Royal Society of Chemistry; (**B**) reproduced from Ref. [49] with permission from the Royal Society of Chemistry.

Qiao et al. designed a fluorescence resonance energy transfer (FRET) nanoprobe using MOFs for the detection of *S. aureus* [48] (Figure 3A). The zirconium (Zr)-based MOFs were used to encapsulate blue-emitting 7-hydroxycoumarin-4-acetic acid (HCAA) and then functioned as an energy donor. By calculating the results, they achieved the detection of *S. aureus* within a dynamic range of 1.05 × 10^3^–1.05 × 10^7^ CFU/mL and a detection limit of 12 CFU/mL. Bhardwaj et al. developed a new luminescent probe for *S. aureus* detection based on the bio-conjugation of an amine functionalized metal–organic framework with an anti-*S. aureus* antibody [49] (Figure 3B). This innovative design of the biosensor allowed the detection of *S. aureus* over a wide concentration range, achieving a notably low limit of detection of 85 CFU/mL.

However, as an emerging crystalline material, MOFs still have some challenges and problems. Due to the complex structure of MOFs, their stability is very low, and they are prone to structural decomposition or ligand bond breakage [66]. In addition, the poor conductivity and low thermal, chemical, and water stability also limit their application [67,68].

### 2.4. Upconversion Nanoparticles

Upconversion nanoparticles (UCNPs) are kinds of special materials with anti-Stokes luminescence processes. While most fluorescent materials are excited by high-energy light to emit low-energy light, upconversion luminescence can be excited by near infrared. This characteristic provides several advantages, including high penetrability and low auto-fluorescence background [69]. UCNPs offer additional benefits such as environmental protection of a cleaner synthetic route, low cost, straightforward detection, and no matrix interference [70,71]. Owing to their low autofluorescence background, deep light penetration, non-toxicity, and minimal photodamage to biological samples, UCNPs have proven to be a universal tool in the past few years [72].

**Figure 4:**
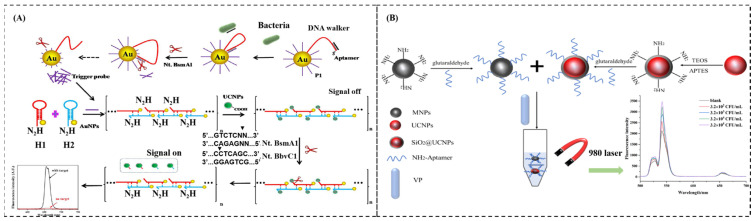
(**A**) The design of a fluorescent aptasensor based on DNA walking and a hybridization chain reaction strategy. (**B**) Schematic illustrations of the detection processes of *Vibrio parahaemolyticus* based on magnetic and upconversion nanoparticles combined with aptamers. (**A**) Adapted with permission from Ref. [50]. Copyright 2024 Elsevier; (**B**) adapted from Ref. [51].

The luminescence mechanism of upconversion can be divided into excited state absorption, energy transfer upconversion, and photon avalanche [73]. Zhang et al. present a new fluorescent aptasensor that integrates DNA walking and a hybridization chain reaction (HCR) to detect *S. aureus* [50] (Figure 4A). The binding of *S. aureus* to the aptamer caused the DNA walker to move along the surface of AuNPs, triggering the separation of the probe and AuNPs, which further triggered the HCR. Therefore, the distance between AuNPs and the upconversion becomes farther away, resulting in the recovery of fluorescence intensity of the upconversion. The limit of detection is 10 CFU/mL, and the detection time is less than 3 h. Song et al. used an aptamer-modified magnetic nanoparticle as a capture probe and an aptamer-modified upconversion nanoparticle a signal probe to capture *Vibrio parahaemolyticus* [51] (Figure 4B). The aptamer-modified magnetic nanoparticle, *Vibrio parahaemolyticus*, and the aptamer-modified upconversion nanoparticle formed a sandwich-like complex, which was rapidly separated from a complex matrix using a magnetic force, and the bacterial concentration was determined by fluorescence intensity analysis. In addition, UCNPs have also been widely used in *Escherichia coli* [52], *Staphylococcus aureus* [53], *Shigella* [54], etc. However, UCNPs still face several challenges [74], including some inherent limitations, such as low quantum yields and narrow absorption cross-sections, which should pay more attention in future research.

### 2.5. Others

Many other materials have also been used for food-borne pathogen detection. Gold nanoclusters (AuNCs) are widely used because of their ultra-small size, tunable emission, size-dependent fluorescence, and good biocompatibility [75]. Additionally, some other 2D nanomaterials, such as manganese dioxide (MnO_2_) nanosheets, are generally used as fluorescence quenchers in fluorescent biosensors [76].

## 3. Fluorescence Sensors

Fluorescence sensors usually consist of two parts: a biosensing (or biorecognition) component and a fluorescence signal. These fluorescence sensors serve as detection tools that convert information regarding a chemical or physical property of the system into a beneficial analytical fluorescence signal [77]. At present, antibodies and aptamers are the two main biosensing components for the specific recognition of food-borne pathogens. Fluorescence signals can be supplied by nanoparticles and fluorescent dyes. However, fluorescent dyes are applied more in the detection of heavy metals, and they have been replaced by fluorescent nanoparticles due to their poisonous and high background fluorescence interference. The components of specific recognition include immunological based, nucleic acid based, and bacteriophage based. Therefore, label-free based fluorescence probes, aptamer-based fluorescence sensors, antigen–antibody-based biosensors, and bacteriophage-based fluorescence sensors were discussed.

### 3.1. Label-Free-Based Fluorescence Probe

**Figure 5:**
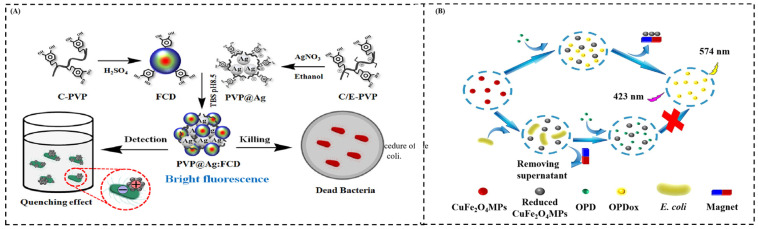
(**A**) Schematic illustration of the principle and procedure of the label-free fluorescent assay for the detection of *E. coli* and *S. aureus*. (**B**) Schematic illustration of the principle and procedure of the label-free fluorescent assay for the detection of *E. coli*. (**A**) Adapted with permission from Ref. [33]. Copyright 2019 Elsevier; (**B**) adapted with permission from Ref. [78]. Copyright 2022 Elsevier.

A label-free based fluorescence probe is a highly sensitive analytical method without any recognition element, such as an antibody or aptamer. At present, some label-free-based fluorescence probes to detect food-borne pathogens have been reported. Sang et al. developed a fluorescence probe consisting of fluorescence carbon dots modified by poly(vinylpyrrolidone) and catechol and silver nanoparticles based on Forster resonance energy transfer for the detection of bacteria without any label [33] (Figure 5A). The fluorescence probe can be recognized through electrostatic binding between the positively charged fluorescence probe and negatively charged bacteria. Furthermore, the fluorescence probe presents excellent bacterial (*E. coli* and *S. aureus*) killing. Fu et al. developed a label-free fluorescent sensor that relies on the competitive reduction in Cu^2+^ in CuFe_2_O_4_ magnetic particles (MPs) by *E. coli* and o-phenylenediamine (OPD) [78] (Figure 5B). In this system, Cu^2+^ in CuFe_2_O_4_ MPs can oxidize OPD to produce 2,3-diaminophenazine (OPDox) with fluorescent properties. The presence of *E. coli* can weaken the oxidative ability of CuFe_2_O_4_ MPs and result in a decrease in the fluorescence of the system. The detection range of *E. coli* is 103–106 CFU/mL, and the detection limit is calculated to be 5.8 × 10^2^ CFU/mL.

### 3.2. Antigen–Antibody-Based Fluorescence Biosensors

Antibodies are large Y-type proteins that are the most commonly used elements in pathogen identification due to their versatility, high sensitivity, and selectivity [79]. They possess remarkable selectivity and affinity for the specific antigen with which they interact [74]. However, the poor stability and high price of antibodies, especially monoclonal antibodies, have become one of the reasons limiting their application [80].

**Figure 6:**
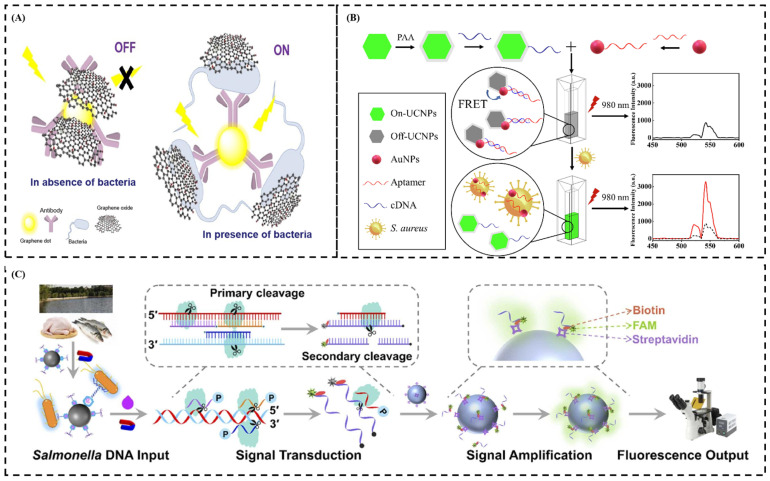
(**A**) Schematic representation of the antibody-GQDs/GO-based biosensor for *Campylobacter jejuni* whole-cell detection. (**B**) Upconversion nanoparticle-based FRET system for the sensitive detection of *S. aureus*. (**C**) Schematic diagram of the biosensor for detecting viable *S. typhimurium* based on bacteriophage. (**A**) Adapted with permission from Ref. [81]. Copyright 2020 Elsevier; (**B**) adapted with permission from Ref. [83]. Copyright 2021 Elsevier; (**C**) adapted with permission from Ref. [84]. Copyright 2024 Elsevier.

Zahra et al. made a fluorescence immunosensor consisting of graphene oxide and antibody-modified graphene dots based on FRET to detect campylobacter jejuni in food samples [81] (Figure 6A). The principle of the fluorescence immunosensor is based on the ability of antibody specificity to bacteria cells. In the present target, the conjugated antibody bacteria caused an inhibition on the π-π interaction between graphene oxide and antibody-modified graphene, leading to the fluorescence recovering through releasing the effect of FRET. Furthermore, the fluorescence immunosensor can finish campylobacter jejuni detection with high sensitivity (LOD = 10 CFU/mL) within 1.5 h. Wang et al. developed an antigen–antibody-based fluorescence biosensor for the detection of *E. coli* O157:H7 [82]. In this research, carbon dots were utilized as fluorescence donors, while covalent organic frameworks served as fluorescence acceptors. An antibody (Ab) specific to *E. coli* O157:H7 was employed to create a CD-Ab-COF immunosensor by linking CDs and COFs. When the antibody was specifically bound with *E. coli* O157:H7, the connection between CDs and COFs was interrupted, resulting in the restoration of carbon dot fluorescence. The sensor exhibited a linear detection range spanning from 0 to 106 CFU/mL, with a limit of detection of 7 CFU/mL.

### 3.3. Aptamer-Based Fluorescence Biosensors

Aptamers are short oligonucleotide molecules (ssDNA or RNA) that typically range from 25 to 90 bases in length and are capable of binding strongly, specifically to target molecules [85,86]. Aptamers can specifically recognize and bind with targets through non-covalent interactions such as hydrogen bonding, van der Waals forces, electrostatic interactions, hydrophobic effects, and π-π stacking [87]. Compared with antibodies, aptamers possess enormous advantages like stability across various temperatures and pH, ease of production, longer shelf life, fast production, and low batch variability, which propell its boom in the field of biosensing.

In aptamer-based fluorescence biosensors, because most targets are non-fluorescent, various fluorescence signal generation strategies are used to detect fluorescence signals. Common fluorescence signal generation strategies include FRET, fluorophore-linked aptamer assays [88], fluorescent light-up aptamers, and fluorescence anisotropy. For example, Ouyang et al. developed an aptamer based on upconversion fluorescence resonance energy transfer for *S. aureus* detection [83]. In this research, the AuNPs were functionalized with aptamers, whereas UCNPs were conjugated with aptamers of complementary DNA (cDNA) (Figure 6B). The complementary base pairing between cDNA and aptamers facilitated the interaction between UCNPs and AuNPs, leading to the quenching of upconversion fluorescence. AuNPs functionalized with aptamers preferentially bound to *S. aureus* and released the UCNPs, resulting in the recovery of UCNP fluorescence.

However, challenges still remain for the practical application of these biosensors, which must be overcome. As food is a complex matrix, the sensitivity and selectivity of the aptamers are affected by the sample conditions, including interfering components, pH, ionic strength, and viscosity [89].

### 3.4. Fluorescence Sensors Based on Bacteriophages

Bacteriophages are viruses of bacteria consisting of DNA or RNA, which can infect host cells and be capable of self-replication within a short period of time. In the beginning, the bacteriophage is used to control bacterial growth. In recent years, a new detection technology based on bacteriophage specificity has emerged. Due to the continuous extension of visualization technology, a novel fluorescent probe based on bacteriophages for the detection of food-borne pathogens obtained success.

Chen et al. used a biotin-expressing T7 bacteriophage combined with avidin-modified FeCo magnetic nanoparticles for the isolation of pathogenic bacteria [90]. The nanoprobe allowed the specific recognition and attachment to *E. coli* cells, and the isolation efficiency was comparable to that of antibody-labeled pathogenic bacteria. Zhao et al. explored a fluorescence biosensor mediated by phage and Clostridium butyricum Argonaute (CbAgo) for the detection of viable Salmonella typhimurium without the need for complicated DNA extraction and amplification procedures [84] (Figure 6C). In this approach, a phage was used to capture viable *S. typhimurium*, while a lysis buffer was used to lyse the *S. typhimurium*. Subsequently, CbAgo can cleave the bacterial DNA to yield target DNA that directs a newly targeted cleavage of fluorescent probes. After that, the resulting fluorescent signal accumulates on the streptavidin-modified single microsphere. This entire detection process was then analyzed and interpreted using machine vision and learning algorithms, allowing for highly sensitive detection of *S. typhimurium* with a limit of detection of 40.5 CFU/mL and a linear range of 50–107 CFU/mL.

The stability of phages at different pH and temperatures makes it an advantageous probe in the field of microbial detection [90]. However, it only targets bacteria that can serve as phage hosts, which limits its widespread application.

## 4. Fluorescence Sensors Combined with Other Techniques

### 4.1. LAMP Combined with Fluorescence Sensors

**Figure 7:**
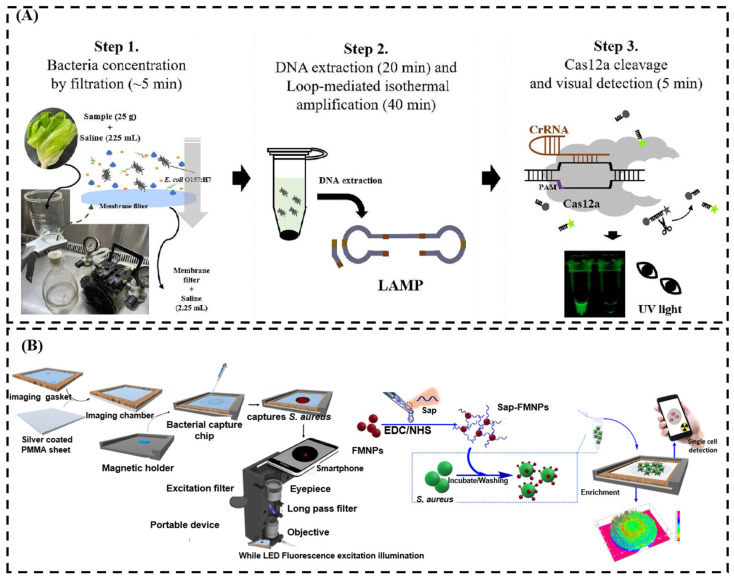
(**A**) Schematic of a visualized detection system using filtration based on LAMP-CRISPR/Cas12a. (**B**) Schematic of detection of *S. aureus* using fluorescence imaging by a smartphone. (**A**) Adapted with permission from Ref. [91]. Copyright 2022 Elsevier; (**B**) adapted with permission from Ref. [92]. Copyright 2018 Elsevier.

Loop-mediated isothermal amplification (LAMP) is a nucleic acid amplification method known for its high specificity and simplicity compared to PCR. Some detection targets can be quantitatively analyzed by analyzing the products in the process. With the development of visualization technology, a new fluorescence probe based on LAMP with fluorescence marks was fabricated. Currently, many fluorescent probes based on LAMP for the detection of food-borne pathogens have been reported. For example, Lee et al. developed a rapid, sensitive, and visual detection of *E. coli* based on CRISPR/Cas12a and LAMP technology. This method is capable of correcting false-negative results produced by LAMP and achieves a detection limit of 1.22 CFU/mL, which was successfully detected without pre-microbial enrichment culture [91] (Figure 7A).

### 4.2. Fluorescence Image Combined with Fluorescence Sensors

Fluorescence image detection technology is a fast and real-time analytical method. Due to different fluorescence materials, fluorescence can be divided into probes consisting of fluorescence nanoparticles and fluorescent dyes combined with a microscope, and some related research work for food-borne detection has been reported. For instance, Sajal et al. developed a quantitative detection for *S. aureus* detection using fluorescence images in peanut milk [92] (Figure 7B). The fluorescence figures were taken by a smartphone camera with a light-emitting diode as the excitation light source. The principle of fluorescence image detection is based on aptamer-functionalized fluorescent magnetic nanoparticles that capture *S. aureus* cells, enabling quantitative analysis through fluorescence imaging. This fluorescence image technology for *S. aureus* detection presents many advantages, including being fast (10 min), easy to operate, having a high sensitivity (LOD = 10 CFU/mL), and having high selectivity [92].

### 4.3. Q-PCR Combined with Fluorescence Sensors

The polymerase chain reaction (PCR) is a technology which can produce multiple copies of a target DNA [93]. The monitoring of food-borne pathogens using PCR can be divided into two types, conventional PCR and qPCR. Conventional PCR Detection is based on stained gel electrophoresis without high specificity. Q-PCR refers to the introduction of fluorescently labeled probes or fluorescent chemicals during PCR amplification. Through the change in fluorescence intensity during cyclic amplification, the DNA sequence in the sample can be quantified.

With the development of detection technology, qPCR as a new fluorescence probe was developed, which has the the disadvantage of low specificity. Additionally, many research works about food-borne pathogens detected by qPCR were reported in recent years. For example, Jun et al. developed a multiplex real-time qPCR technology combined with polymer network carbon nanotubes to detect four bacteria (*P. aeruginosa*, *K. pneumoniae*, *A. baumannii*, *E. coli*) [94]. The four bacteria can be differentially identified in 30 min by this qPCR detection method. Pan et al. present a fast analytical method to detect *E. coli* O157:H7 in food samples based on propidium monoazide combined with droplet PCR [95]. Propidium monoazide is a DNA amplification inhibitor that penetrates into injured cells with compromised membrane integrity. However, this inhibitor is not able to penetrate into viable cells. qPCR technology combined with propidium monoazide finished *E. coli* detection in more than 4.5 h with high sensitivity (LOD = 1 CFU/mL) and without false positive interference. The above successful food-borne pathogen detection examples show qPCR’s superiority of high sensitivity and high selectivity as a fluorescence probe.

## 5. Conclusions and Challenges

In order to better cope with the challenges posed by food-borne pathogens, this review summarized the fluorescence materials and different kinds of fluorescence sensors in the detection of food-borne pathogens. We highlighted the optical properties of different fluorescence materials. Additionally, to present an in-depth analysis of the utility of various fluorescence assays for food safety, their detection line, assay time, and linear detection range values were discussed. The QDs have strong fluorescence emission efficiency, and the structure of QDs makes it easy for them to be modified by biological molecules, like antibodies and aptamers. However, QDs are toxic, which greatly limits their applications. Compared to conventional quantum dots, tCDs have the advantages of low toxicity and high water stability. MOFs have advantages, including a high specific surface area, controllable pore structures, and significant thermal stability, but they are poor in stability. UCNPs have the advantages of low toxicity and unique luminescent properties, which are applied in multiple detections of pathogens. Moreover, different fluorescence biosensors are reviewed according to the biosensing components, which show great potential in food-borne pathogen detection.

Although nanomaterial-based fluorescent assays have advantages compared to traditional detection methods, they still have some limitations. (1) They have high technical requirements and require special equipment and technical personnel to operate. (2) They have a high cost. Fluorescent nanomaterials and detection equipment are usually expensive, which limit their application scenarios and promotion scope. (3) Limitations and interferences can arise when using fluorescent sensors with different food matrices, which may lead to false positives or false negatives. Based on the above summary, the following points should be considered. (1) With the development of miniaturized hardware technology, the limitations of traditional detection procedures finished by professionals in the laboratory no longer exist, and more portable detection devices can be applied. (2) Smart devices, such as smartphones, should be combined with fluorescence sensors to create a new intelligent fluorescence rapid detection platform. (3) Suitable methods and devices that meet the urgent need for efficient on-site testing of food samples and the environment should be developed. (4) With the diversity and complexity of pathogen contamination in food substrates, a suitable selection of biometric elements and nanomaterials to detect multiple bacteria is becoming more important. (5) It is necessary to enhance the resistance of fluorescent nanomaterials to harsh environments to avoid the influence of environmental factors on fluorescence intensity and fluorescence lifetime.

## Figures and Tables

**Table 1:** List of the detailed detection methods for food-borne pathogens.

Method		Food-Borne Pathogens	Assays Time	Detection Limit (CFU/mL)	Potential Limitations	References
Biosensors	Electrochemical biosensor	*Staphylococcus aureus*	>90 min	2	Complex manufacture procedure and no anti-interference in real sample detection application	[24]
	Electrochemical biosensor	*Escherichia coli* O26	24 h	0.04		[25]
	SERS biosensor	*E. coli* O157:H7	>30 min	1	Poor stability	[26]
	SERS biosensor	*Staphylococcus aureus*	>15 min	1.5		[27]
	LSPR biosensor	*E. coli* O157:H7	<2 h	10	Narrow detection linear range and poor stability	[28]
	Fluorescence biosensor	*Staphylococcus aureus*	1.5 h	40	-	[29]
	Fluorescence biosensor	*Staphylococcus aureus*	>75 min	-	-	[30]
Chemical sensor	Dopamine modification fluorescence sensor	*Staphylococcus aureus*	>80 min	103	No selectivity	[31]
	pH-based fluorescence sensor	*Escherichia coli* and *Staphylococcus aureus*	6–8	21 and 33	Long time	[32]
	Poly(vinylpyrrolidone) (PVP) and catechol modification fluorescence sensor	*E. coli* and *S. aureus*	>1 h	10 and 10	No selectivity	[33]
PCR	qPCR	*E. coli* O157:H7	-	1	High cost	[34]
	dPCR	*Vibrio parahaemolyticus*	-	15	Complex manufacturing procedure	[3]
Device	Fluorescence based	*E. coli* K-12	50 min	3.4 × 105	Low sensitivity	[35]

**Table 2:** Several fluorescence analysis methods based on different fluorescence materials for food-borne pathogen detection.

Bacterium	Fluorescence Materials	Assay Time	LOD (CFU/mL)	Samples	References
*Salmonella typhimurium*	QDs	<2 h	1	milk	[42]
*E. coli* O157: H7 *Salmonella typhimurium*	QDs@MnO_2_	<2 h	15 and 40	egg	[43]
*E. coli* O157: H7	CDs	<10 min	10	milk	[44]
*Staphylococcus aureus*	CDs@BONs	>15 min	1.5	-	[45]
*Helicobacter pylori*	CDs	-	10	lettuce	[46]
*Salmonella*	dSiO_2_@CDs	-	36	milk	[47]
*Staphylococcus aureus*	MOFs	-	12	juice	[48]
*Staphylococcus aureus*	MOFs	-	85	cream pastry	[49]
*Staphylococcus aureus*	UCNPs	<3 h	10	honey	[50]
*Vibrio parahaemolyticus*	UCNPs	-	4.4	-	[51]
*E. coli* O157: H7	UCNPs@NB	<1.5 h	33	chicken	[52]
*Staphylococcus aureus*	MNPs@GO-UCNPs	-	13	chicken	[53]
*Shigella*	UCNPs	<1 h	30	chicken	[54]

## Data Availability

Not applicable.
